# Emerging Infectious Diseases: Trends and Issues, 2nd Edition

**DOI:** 10.3201/eid1402.071424

**Published:** 2008-02

**Authors:** Laura D. Kramer, Elizabeth Kauffman

**Affiliations:** * Wadsworth Center–New York State Department of Health, Albany, New York, USA

This second edition of Emerging Infectious Diseases: Trends and Issues, edited by F.R. Lashley and J.D. Durham, appearing 5 years after the first edition, has added several important diseases such as severe acute respiratory syndrome, avian influenza, and monkeypox. The authors also have updated the chapters included in the first edition. At nearly 600 pages, the book gives a broad overview of 22 emerging diseases with mention of many others. Topics such as antimicrobial drug resistance, bioterrorism, and travel-associated issues also are covered. Persons interested in having a bedside companion about microbes out there in the world waiting to infect humans will enjoy this book, and medical personnel should find it useful. Many chapters begin with a clinical case history, which captures the reader’s attention and transports him or her into the mindset of a clinician. Yet the book by no means can serve as a diagnostic manual. Rather, it provides basic information on diseases and issues that should be kept in mind in diagnostics, such as behavioral, cultural, and environmental factors that influence the likelihood of infection with particular pathogens.

The broad scope of topics covered is impressive, but the book does not cover any specific disease rigorously. To avoid inaccuracies, the submitted manuscript would have benefited from review by scientists with current expertise regarding specific pathogens. For instance, it is stated that West Nile virus–infected horses maintain “moderate to high levels of virus,” whereas recent research indicates low transient viremia in equines that cannot infect mosquitoes (*1*). In addition, some statements may be misleading, such as “high levels of the [West Nile] virus in birds may persist for long periods of time (20–100 days); thus, migratory birds are implicated in the introduction of WNV.” Recent studies indicate that high levels of viral RNA but low levels of infectious virus have been recovered from birds up to 6 weeks after infection (*2*,*3*). The epidemiologic significance of these findings is not clear.

Additionally, other concepts are poorly explained, e.g., the discussions on dengue secondary infections and authochthonous transmission. Broad, sweeping statements are made, which by their nature are inaccurate, such as the statement in the introduction that Ebola “came and disappeared.” If only that were so! Also, some important emerging/reemerging diseases are not covered in detail, such as chikungunya, or at all, such as Japanese encephalitis. More problems occur with the glossary, which, for example, defines an arthropod as a “vector belonging to the phylum Arthropoda that transmits an organism from 1 host to another.” This is more a definition of an “arthropod vector” since not all arthropods are vectors. Therefore, scientists, especially those involved in public health, may not find their particular areas of expertise described satisfactorily, but they will find in many areas outside their immediate field of knowledge a useful compact description that addresses the highlights of a particular disease.

Putting these complaints aside, the book serves well as a point of introduction to the major diseases described. The bibliographies have been strengthened since the first edition and are useful guides for further in-depth inquiry. The 4 appendixes are a strong point. Appendix D provides information on available resources for learning more about specific diseases, appendixes A and B list and describe emerging/reemerging infectious diseases by organism and by modes of transmission, and appendix C lists measures to prevent these diseases. Overall, we recommend this book to anyone who wants an introduction to the public health aspects of emerging diseases.
